# Fertility and Growth Traits, Some Body Measurements, and Morphological Characteristics of Kars Shepherd Dogs

**DOI:** 10.1002/vms3.70660

**Published:** 2025-10-18

**Authors:** Fikret Bektaşoğlu, Turgut Kırmızıbayrak

**Affiliations:** ^1^ Department of Animal Breeding and Husbandry, Faculty of Veterinary Medicine Kafkas University Kars Türkiye

**Keywords:** body measurements, fertility traits, growth traits, Kars Shepherd dogs

## Abstract

This study aimed to determine the fertility and growth traits, some body measurements, and morphological characteristics of Kars Shepherd dogs. A total of 21 bitches were used to determine fertility traits and 57 puppies were used for growth traits, some body measurements, and some important morphological characteristics. Body measurements of the puppies were taken at 45th, 60th, and 75th days of age, and subsequently on a monthly basis from 3 to 12 months of age. The mean age at first estrus was 427 days, with an average estrous cycle length of 234.7 days, the duration of mating was 18.9 min, and the gestation period averaged 62.7 days. Both estrus rate and whelping rate values were 100%, while the stillbirth rate was 27.9%, and the average litter size was 5.03. Means of birth weight, live weight at the 3rd, 6th and 12th months were 0.51, 12.10, 21.89 and 40.63 kg, respectively. The mean of head circumference was 51.92 cm, muzzle circumference was 30.62 cm, chest circumference was 79.77 cm, chest depth was 26.23 cm, height at withers was 69.34 cm, height at rump was 68.58 cm, body length was 72.04 cm and front wrist circumference was 13.70 cm in Kars Shepherd dog puppies at 12 months of age. The average age at first opening of the eyelids, age at first opening of the external ear canal, age at first eruption of deciduous teeth, and age at the eruption of first permanent teeth were 14.02, 16.95, 25.21 days and 12.51 weeks for Kars Shepherd puppies, respectively. These findings will increase our knowledge about the Kars Shepherd dog, about which there is not enough information, and will be a source of literature for future studies.

## Graphical abstract

The animals were mated, and their care and nutrition were maintained regularly until parturition. Beginning at birth, body measurements were taken and recorded up to 12 months of age. In addition, the animals were visually documented through periodic photographic records.

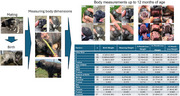

## 1 Introduction

Dogs have an important role in human history and have accompanied humans for thousands of years. Archaeological and genetic studies have revealed that dogs were the first animal species domesticated by humans (Savolainen et al. 2002). Today, there are numerous dog breeds worldwide, which are classified into various groups by relevant organizations. One of the most important dog groups is the shepherd dogs (Federation Cynologique Internationale 2023). Breeders generally used Shepherd dogs to manage herds and protect livestock against external threats (United Kennel Club 2023).

In Türkiye, dogs have historically been bred to guard rural areas, housing facilities and herds of livestock. For these reasons, Shepherd dogs are the most recognized and significant native dog genotypes in Türkiye. Among Turkish Shepherd dog breeds, one of the most important is the Kars Shepherd dog breed. This breed shares similarities with the Caucasian Shepherd dog breed and was first reported by David Nelson at the Konya International Shepherd Dog Symposium in 1996 (Nelson 1996). Afterward, this breed was officially registered as a Kars Turkish Shepherd dog by the Military Veterinary School and Training Center Command in Gemlik, Bursa, Türkiye, following an application to the Turkish Patent and Trademark Office in 2002 (Turkish Patent and Trademark Office 2023).

The coat colour of the Kars Shepherd dog is predominantly grey, with lighter white or yellowish markings around the tail, legs, underbelly and face. They generally have a black mask on their face, covering the muzzle and nose. The head is generally dark in some individuals, the area around the eyes is distinctly light white or yellowish in some, and the ears are dark grey or black in almost all individuals. The head structure resembles that of a bear's head. The coat is generally long, while short haired varieties also exist. The fur is significantly longer on the back of the legs than on the other parts of the body (Kirmizibayrak 2004). Despite their historical and genetic significance, Kars Shepherd dogs lack substantial scientific documentation on important breed traits. Although some studies have addressed their general appearance and characteristics, (Kirmizibayrak 2004; Erdoğan et al. 2012; Tepeli et al. 2017), there exists a significant gap regarding their fertility traits, growth patterns and detailed physical measurements. Therefore, a thorough study was required to establish baseline physiological and morphological characteristics of Kars Shepherd dogs, which can establish future breeding and conservation initiatives.

This study aimed to determine the fertility traits, growth patterns, certain physical measurements and morphological features of Kars Shepherd Dogs. The results of this investigation will enhance our understanding and facilitate the conservation of Kars Shepherd dogs as a valuable domestic genetic resource in Türkiye.

## 2 Materials and Methods

### 2.1 Ethical Approval

This study was carried out at the breeding facility at the Kafkas Education‐Research and Experimental Farm, Faculty of Veterinary Medicine, Kafkas University, Kars, Türkiye. Ethical approval was obtained from the Kafkas University Animal Experiments Local Ethics Committee for compliance with ethical guidelines under decision number KAU‐HADYEK/2021‐146.

### 2.2 Animal Material

The breeding material of the study consisted of 11 female and 5 male Kars Shepherd dogs, aged 3–8 years, and 57 puppies produced through their mating. Body measurements of the puppies were taken at 45th, 60th and 75th days of age, and subsequently on a monthly basis from 3 to 12 months of age.

### 2.3 Feeding Management

The feeding regimen was designed to meet the nutritional requirements of adult dogs and growing pups (FEDIAF 2023). The diet composition for adult dogs comprised 27% crude protein, 370 kcal/100 g energy, 16% crude fat, 2.5% crude fibre, 6.5% crude ash, 1.35% calcium, 1.00% phosphorus and 0.30% sodium. New‐born puppies were fed with maternal milk during the suckling period following colostrum phase. After the suckling period, puppies were gradually transitioned to a solid diet using puppy food moistened with cow milk. This transition period lasted for 15 days, during which the puppies were fed ad libitum in four meals per day. The nutritional composition of the puppy food consisted of 26% crude protein, 420 kcal/100 g energy, 13% crude fat, 10% moisture, 8% crude ash, 4% crude fibre, 2% calcium, 1% phosphorus, 0.5% sodium, 15.000 IU/kg vitamin A, 1.250 IU/kg vitamin D, 100 mg/kg vitamin E and C 150 mg/kg vitamin. All dogs included in the study were subjected to a preventive health care program (Day et al. 2016).

### 2.4 Reproductive Performance, Growth, and Some Morphological Characteristics

For the evaluation of reproductive traits, data were collected from 21 female dogs, comprising 10 young primiparous individuals and 11 adult multiparous dogs that had reached sexual maturity. To assess survival rates, records of 116 live‐born puppies (69 males and 47 females) were analysed. To determine live weight and specific body measurements, data from 57 puppies (47 males and 10 females) were utilized.

### 2.5 Statistical Analysis

Statistical analysis was conducted using the SPSS software package (version 26.0, Chicago, IL, USA). The normality of data distribution across groups based on sex, litter size, birth year and birth season was tested using the Shapiro–Wilk test. For pairwise comparisons, an independent samples *t*‐test was applied. The effects of sex, litter size, year and season on the live weight were determined using the General Linear Model. Post‐hoc comparisons were performed using Duncan's multiple‐range test. Survival rates by sex were analysed using the chi‐square test. The data obtained for the examined traits were presented as mean ± standard error of mean (SEM). Differences between groups were considered statistically significant at *p* ≤ 0.05.

## 3 Results

### 3.1 Reproductive Characteristics

In this study, the means of pubertal estrus, inter‐estrus intervals, the duration of proestrus, the duration of estrus, gestation period and duration of mating for Kars Shepherd dogs were found 427.01, 234.72, 7.12, 6.42, 62.78 days and 18.98 min, respectively (Table 1). The highest estrus occurrence in female dogs was recorded in autumn (September–November) at 37.50%, followed by spring (March–May) at 34.37%. Lower estrus rates were observed in winter (December–February) at 21.87%, while the lowest in summer (June–August) at 6.25%. In bitches, the oestrus rate was 100%, the stillbirth rate was 27.95% and the average litter size was 5.03.

**TABLE 1:** Some reproductive characteristics of Kars Shepherd dogs.

Traits		*n*	X̄ ± Sx̄	Min.	Max.
Pubertal estrus (days)		10	427.01 ± 2.04	420	437
Inter‐estrus intervals (days)		11	234.72 ± 5.42	221	274
The duration of proestrus (days)		21	7.12 ± 0.21	5	10
The duration of estrus (days)		21	6.42 ± 0.22	5	10
Duration of mating (min.)		21	18.98 ± 0.33	14	28
Gestation period (days)		21	62.78 ± 0.25	60	65
The mean litter size (number of births = 32)	Female	76	2.37 ± 0.20	1	5
	Male	85	2.65 ± 0.17	0	4
	Total	161	5.03 ± 0.17	4	8
Number of stillbirth puppies per litter (number of births = 32)	Female	29	0.90 ± 0.12	0	3
	Male	16	0.50 ± 0.13	0	3
	Total	45	1.40 ± 0.12	0	3

### 3.2 Survival Rates of Puppies

In this study, for survival rate determination, 101 puppies (60 males and 41 females) survived until weaning age, while 98 puppies (58 males and 40 females) survived until 2 months of age. Survival rates at weaning age and two months old were found 87.1 and 84.5 % respectively Table 2.

**TABLE 2:** Survival rates of Kars Shepherd dog puppies.

Age period	*n*	Male (%)	Female (%)	*χ* ^2^	*p* value	General (%)
Birth (Day 0)—weaning (Day 45)	101	87.0	87.2	0.002	0.965	87.1
Birth (Day 0) ‐ 2 months of age	98	84.1	85.1	0.023	0.878	84.5

### 3.3 Live Weight of Puppies

The average live weights of the Kars Shepherd dog puppies according to age and sex are presented in Table 3. The effects of litter size, year of birth, season of birth and sex on live weight at various growth periods are given in Table 4. The average birth weight, weaning weight, live weight at 3, 6 and 12 months of age were 0.51, 4.31, 12.10, 21.89 and 40.63 kg, respectively.

**TABLE 3:** Mean live weights (kg) of male (*n* = 47) and female (*n* = 10) puppies of Kars Shepherd dog at different ages.

Age	Male X̄ ± Sx̄	Female X̄ ± Sx̄	*p* value	General X̄ ± Sx̄
1st week	0.77 ± 0.01	0.72 ± 0.01	0.010	0.76 ± 0.008
2nd week	1.36 ± 0.01	1.24 ± 0.02	<0.001	1.34 ± 0.01
3rd week	1.88 ± 0.01	1.81 ± 0.02	0.028	1.87 ± 0.01
4th week	2.86 ± 0.02	2.40 ± 0.13	0.007	2.78 ± 0.03
8th week	6.84 ± 0.07	6.45 ± 0.09	0.019	6.77 ± 0.06
10th week	8.75 ± 0.09	8.21 ± 0.14	0.015	8.66 ± 0.08
4th month	15.02 ± 0.20	14.00 ± 0.21	0.001	14.84 ± 0.17
5th month	18.27 ± 0.21	17.30 ± 0.34	0.033	18.10 ± 0.17
7th month	28.04 ± 0.33	25.85 ± 026	<0.001	27.66 ± 0.29
8th month	32.65 ± 0.27	30.20 ± 0.46	<0.001	32.22 ± 0.26
9th month	35.37 ± 0.26	33.00 ± 0.44	<0.001	34.96 ± 0.25
10th month	37.65 ± 0.27	34.95 ± 0.43	<0.001	37.17 ± 0.26
11th month	39.66 ± 0.29	36.15 ± 0.45	<0.001	39.04 ± 0.30

**TABLE 4:** Live weights (kg) of Kars Shepherd dog puppies at various ges of growth (X̄ ± Sx̄).

Factors	*n*	Birth weight	Weaning weight	3‐Month Live Weight	6‐Month Live Weight	12‐Month Live Weight
**Litter Size**						
4	16	0.520 ± 0.004^a^	4.22 ± 0.10^a^	12.13 ± 0.19	21.86 ± 0.34	41.37 ± 0.67
5	33	0.509 ± 0.005^ab^	4.34 ± 0.05^b^	12.08 ± 0.23	21.83 ± 0.31	40.31 ± 0.47
6	8	0.498 ± 0.006^b^	4.39 ± 0.07^b^	12.14 ± 0.83	22.18 ± 0.83	40.45 ± 0.77
*P* value	—	0.002	0.012	0.848	0.871	0.280
**Year of Birth**						
2021	20	0.506 ± 0.005^a^	4.24 ± 0.08	11.35 ± 0.18	21.56 ± 0.25	38.54 ± 0.43
2022	37	0.513 ± 0.004^b^	4.35 ± 0.04	12.51 ± 0.23	22.07 ± 0.33	41.75 ± 0.37
*P* value	—	0.040	0.146	0.215	0.689	0.849
**Season of Birth**						
Spring	22	0.512 ± 0.005	4.37 ± 0.06	12.64 ± 0.16	22.08 ± 0.38	42.88 ± 0.35^a^
Summer	35	0.509 ± 0.004	4.28 ± 0.05	11.76 ± 0.26	21.77 ± 0.30	39.21 ± 0.35^b^
*P* value	—	0.053	0.275	0.714	0.796	<0.001
**Sex**						
Male	47	0.514 ± 0.003	4.37 ± 0.03^a^	12.33 ± 0.20	22.12 ± 0.26^a^	41.31 ± 0.33^a^
Female	10	0.490 ± 0.007	4.03 ± 0.13^b^	11.02 ± 0.21	20.80 ± 0.39^b^	37.40 ± 0.56^b^
*P* value	—	0.355	<0.001	0.291	0.040	0.003
**General**	57	0.510 ± 0.003	4.31 ± 0.03	12.10 ± 0.18	21.89 ± 0.23	40.63 ± 0.35

^a,b^ Differences between means with different letters within some groups are statistically significant (*p *< 0.005).

### 3.4 Body Measurements of Puppies

The average body measurements of Kars Shepherd dog puppies at different age groups are given in Table 5.

**TABLE 5:** Body measurement (cm) of male (*n* = 47) and female (*n* = 10) Kars Shepherd dog puppies at different ages (X̄ ± Sx̄).

Traits	Sex	45 days	60 days	75 days	3 months	4 months	5 months	6 months	7 months	8 months	9 months	10 months	11 months	12 months
Head Length	Male	15.04 ± 0.16	15.67 ± 0.17	16.21 ± 0.19	17.06 ± 0.20	18.33 ± 0.22	19.57 ± 0.23	21.35 ± 0.24	22.37 ± 0.24	23.84 ± 0.27	25.42 ± 0.22	26.23 ± 0.20	26.72 ± 0.19	27.22 ± 0.18
	Female	13.80 ± 0.17	14.50 ± 0.18	15.35 ± 0.08	16.10 ± 0.10	17.45 ± 0.20	18.90 ± 0.30	21.10 ± 0.45	22.65 ± 0.45	23.65 ± 0.37	24.60 ± 0.26	25.25 ± 0.23	25.85 ± 0.20	26.30 ± 0.23
	*p* value	<0.001	0.003	<0.001	<0.001	0.006	0.200	0.655	0.616	0.756	0.111	0.033	0.003	0.023
	General	14.82 ± 0.15	15.46 ± 0.15	16.06 ± 0.16	16.89 ± 0.17	18.17 ± 0.18	19.45 ± 0.19	21.30 ± 0.21	22.42 ± 0.20	23.80 ± 0.23	25.27 ± 0.19	26.06 ± 0.17	26.57 ± 0.16	27.06 ± 0.15
Face Length	Male	4.92 ± 0.13	5.48 ± 0.11	5.77 ± 0.08	6.60 ± 0.07	7.45 ± 0.07	8.05 ± 0.07	8.54 ± 0.07	9.14 ± 0.09	9.51 ± 0.10	9.87 ± 0.09	9.91 ± 0.08	10.20 ± 0.09	10.69 ± 0.08
	Female	5.45 ± 0.16	5.75 ± 0.23	6.10 ± 0.23	6.50 ± 0.22	7.30 ± 0.17	8.05 ± 0.16	8.60 ± 0.12	9.35 ± 0.08	9.60 ± 0.07	9.65 ± 0.08	9.75 ± 0.11	10.05 ± 0.09	10.40 ± 0.12
	*p* value	0.017	0.337	0.132	0.657	0.338	0.986	0.739	0.094	0.475	0.064	0.365	0.243	0.065
	General	5.01 ± 0.11	5.53 ± 0.10	5.83 ± 0.08	6.58 ± 0.07	7.42 ± 0.06	8.05 ± 0.06	8.55 ± 0.06	9.18 ± 0.07	9.52 ± 0.09	9.83 ± 0.07	9.88 ± 0.07	10.17 ± 0.08	10.64 ± 0.07
Head Width	Male	7.86 ± 0.07	8.22 ± 0.08	8.36 ± 0.07	8.77 ± 0.08	9.27 ± 0.08	9.78 ± 0.08	10.54 ± 0.07	11.53 ± 0.06	12.18 ± 0.06	12.57 ± 0.07	12.78 ± 0.06	13.09 ± 0.07	13.48 ± 0.07
	Female	6.80 ± 0.13	7.20 ± 0.17	7.75 ± 0.17	8.05 ± 0.14	8.55 ± 0.14	9.15 ± 0.13	10.00 ± 0.15	11.00 ± 0.15	11.65 ± 0.13	11.90 ± 0.12	12.20 ± 0.13	12.35 ± 0.08	12.65 ± 0.11
	*p* value	<0.001	<0.001	0.001	<0.001	<0.001	0.001	0.001	0.001	<0.001	<0.001	<0.001	<0.001	<0.001
	General	7.67 ± 0.08	8.04 ± 0.09	8.25 ± 0.07	8.64 ± 0.08	9.14 ± 0.08	9.67 ± 0.07	10.44 ± 0.07	11.43 ± 0.06	12.08 ± 0.06	12.45 ± 0.07	12.68 ± 0.06	12.96 ± 0.07	13.34 ± 0.08
Head Circum ference	Male	28.54 ± 0.16	29.51 ± 0.14	30.18 ± 0.15	31.91 ± 0.16	34.61 ± 0.15	37.46 ± 0.18	40.97 ± 0.17	43.53 ± 0.19	45.94 ± 0.22	48.23 ± 0.32	49.48 ± 0.32	51.24 ± 0.34	52.56 ± 0.40
	Female	26.95 ± 0.36	28.00 ± 0.33	29.10 ± 0.32	30.35 ± 0.39	32.75 ± 0.38	35.50 ± 0.34	38.45 ± 0.41	41.40 ± 0.39	43.70 ± 0.40	45.45 ± 0.41	46.90 ± 0.40	48.00 ± 0.35	48.90 ± 0.32
	*p* value	<0.001	<0.001	0.005	<0.001	<0.001	<0.001	<0.001	<0.001	<0.001	<0.001	0.001	<0.001	<0.001
	General	28.26 ± 0.17	29.24 ± 0.15	29.99 ± 0.15	31.64 ± 0.17	34.28 ± 0.17	37.12 ± 0.19	40.53 ± 0.20	43.15 ± 0.20	45.55 ± 0.22	47.74 ± 0.31	49.03 ± 0.30	50.67 ± 0.33	51.92 ± 0.38
Ear Length	Male	10.42 ± 0.13	10.90 ± 0.13	10.93 ± 0.12	11.07 ± 0.12	11.88 ± 0.13	12.42 ± 0.13	13.43 ± 0.13	14.44 ± 0.14	15.76 ± 0.15	16.36 ± 0.13	16.76 ± 0.14	16.86 ± 0.13	17.09 ± 0.11
	Female	10.00 ± 0.24	10.55 ± 0.17	10.75 ± 0.19	11.05 ± 0.14	11.60 ± 0.10	12.05 ± 0.14	12.85 ± 0.11	13.80 ± 0.11	14.35 ± 0.08	14.85 ± 0.17	15.00 ± 0.15	15.20 ± 0.13	15.40 ± 0.10
	*p* value	0.152	0.119	0.511	0.894	0.097	0.056	0.001	0.001	<0.001	<0.001	<0.001	<0.001	<0.001
	General	10.35 ± 0.11	10.84 ± 0.11	10.90 ± 0.11	11.07 ± 0.10	11.83 ± 0.11	12.35 ± 0.11	13.33 ± 0.12	14.33 ± 0.12	15.51 ± 0.14	16.09 ± 0.14	16.45 ± 0.15	16.57 ± 0.14	16.79 ± 0.13
Ear Width	Male	7.47 ± 0.07	7.92 ± 0.08	7.97 ± 0.07	8.05 ± 0.07	8.48 ± 0.07	9.00 ± 0.07	9.94 ± 0.08	10.90 ± 0.09	11.39 ± 0.09	11.50 ± 0.08	11.89 ± 0.09	11.98 ± 0.08	12.25 ± 0.08
	Female	6.80 ± 0.15	7.10 ± 0.10	7.15 ± 0.10	7.50 ± 0.07	7.70 ± 0.11	8.35 ± 0.15	8.85 ± 0.20	9.90 ± 0.24	10.45 ± 0.25	11.00 ± 0.31	11.20 ± 0.33	11.25 ± 0.31	11.50 ± 0.31
	*p* value	<0.001	<0.001	<0.001	<0.001	<0.001	<0.001	<0.001	<0.001	<0.001	0.032	0.007	0.001	0.038
	General	7.35 ± 0.07	7.78 ± 0.08	7.83 ± 0.08	7.95 ± 0.07	8.35 ± 0.07	8.88 ± 0.07	9.75 ± 0.09	10.72 ± 0.10	11.22 ± 0.10	11.41 ± 0.09	11.77 ± 0.10	11.85 ± 0.09	12.12 ± 0.09
Traits	Sex	45 days	60 days	75 days	3 months	4 months	5 months	6 months	7 months	8 months	9 months	10 months	11 months	12 months
Distance between Ears	Male	7.44 ± 0.08	7.90 ± 0.09	8.56 ± 0.10	8.91 ± 0.09	9.44 ± 0.09	9.94 ± 0.08	10.45 ± 0.08	11.36 ± 0.10	11.86 ± 0.10	12.37 ± 0.10	12.65 ± 0.11	12.94 ± 0.09	13.10 ± 0.08
	Female	7.10 ± 0.15	7.30 ± 0.13	7.40 ± 0.12	7.65 ± 0.11	8.20 ± 0.15	8.70 ± 0.15	9.20 ± 0.13	10.25 ± 0.13	10.65 ± 0.11	11.35 ± 0.21	11.80 ± 0.19	11.90 ± 0.18	12.20 ± 0.20
	*p* value	0.075	0.001	<0.001	<0.001	<0.001	<0.001	<0.001	<0.001	<0.001	<0.001	0.002	<0.001	<0.001
	General	7.38 ± 0.07	7.79 ± 0.08	8.35 ± 0.11	8.69 ± 0.10	9.22 ± 0.10	9.72 ± 0.10	10.23 ± 0.10	11.16 ± 0.10	11.64 ± 0.11	12.19 ± 0.10	12.50 ± 0.11	12.76 ± 0.10	12.94 ± 0.09
Chest Circumference	Male	38.62 ± 0.21	39.60 ± 0.77	41.87 ± 0.23	44.39 ± 0.21	48.89 ± 0.24	56.69 ± 0.24	63.86 ± 0.22	67.96 ± 0.23	71.86 ± 0.24	75.31 ± 0.22	78.16 ± 0.22	79.25 ± 0.23	80.17 ± 0.20
	Female	37.10 ± 0.23	39.35 ± 0.20	40.70 ± 0.25	43.15 ± 0.17	47.00 ± 0.17	52.45 ± 0.14	58.30 ± 0.13	64.40 ± 0.15	68.25 ± 0.18	71.85 ± 0.28	74.15 ± 0.31	76.25 ± 0.40	77.09 ± 0.32
	*p* value	<0.001	0.003	0.002	<0.001	<0.001	<0.001	<0.001	<0.001	<0.001	<0.001	<0.001	<0.001	<0.001
	General	38.35 ± 0.19	39.56 ± 0.64	41.66 ± 0.20	44.17 ± 0.19	48.56 ± 0.22	55.94 ± 0.30	62.88 ± 0.34	67.34 ± 0.26	71.22 ± 0.27	74.71 ± 0.25	77.45 ± 0.27	78.72 ± 0.25	79.77 ± 0.21
Leg Length	Male	14.87 ± 0.15	18.80 ± 0.15	22.35 ± 0.13	24.58 ± 0.15	26.60 ± 0.18	30.28 ± 0.18	32.13 ± 0.20	34.98 ± 0.18	36.97 ± 0.20	38.58 ± 0.19	40.47 ± 0.18	42.01 ± 0.18	43.09 ± 0.19
	Female	14.60 ± 0.19	16.45 ± 0.19	18.05 ± 0.19	19.85 ± 0.17	23.35 ± 0.25	26.50 ± 0.20	29.05 ± 0.27	32.50 ± 0.30	35.05 ± 0.28	37.05 ± 0.20	38.35 ± 0.20	39.10 ± 0.21	39.85 ± 0.22
	*p* value	0.425	<0.001	<0.001	<0.001	<0.001	<0.001	<0.001	<0.001	<0.001	<0.001	<0.001	<0.001	<0.001
	General	14.82 ± 0.13	18.39 ± 0.18	21.59 ± 0.24	23.75 ± 0.27	26.03 ± 0.23	29.62 ± 0.24	31.59 ± 0.23	34.55 ± 0.20	36.64 ± 0.20	38.31 ± 0.18	40.10 ± 0.19	41.50 ± 0.21	42.52 ± 0.23
Front Wrist Circumference	Male	8.97 ± 0.19	9.65 ± 0.19	9.90 ± 0.20	9.98 ± 0.19	10.01 ± 0.19	10.15 ± 0.15	11.30 ± 0.17	12.65 ± 0.20	13.15 ± 0.22	13.35 ± 0.24	13.50 ± 0.24	13.80 ± 0.29	14.10 ± 0.26
	Female	7.40 ± 0.12	7.65 ± 0.11	8.10 ± 0.07	8.40 ± 0.07	9.20 ± 0.11	10.12 ± 0.18	10.51 ± 0.18	11.90 ± 0.19	12.45 ± 0.19	12.91 ± 0.20	13.32 ± 0.19	13.44 ± 0.19	13.61 ± 0.19
	*p* value	<0.001	<0.001	<0.001	<0.001	<0.001	0.012	0.006	0.012	0.027	0.171	0.583	0.325	0.257
	General	8.70 ± 0.18	9.30 ± 0.19	9.58 ± 0.19	9.71 ± 0.18	9.86 ± 0.16	10.13 ± 0.15	10.64 ± 0.16	12.03 ± 0.17	12.57 ± 0.17	12.99 ± 0.17	13.35 ± 0.17	13.50 ± 0.17	13.70 ± 0.16
Rear Wrist Circumference	Male	8.97 ± 0.19	9.40 ± 0.20	9.44 ± 0.19	9.46 ± 0.19	9.47 ± 0.19	9.70 ± 0.18	10.65 ± 0.15	11.90 ± 0.21	12.55 ± 0.24	12.80 ± 0.17	13.05 ± 0.23	13.25 ± 0.23	13.40 ± 0.21
	Female	7.10 ± 0.10	7.45 ± 0.14	7.85 ± 0.11	8.15 ± 0.08	8.55 ± 0.05	9.55 ± 0.14	10.00 ± 0.19	10.95 ± 0.19	11.44 ± 0.19	12.37 ± 0.20	12.85 ± 0.20	12.93 ± 0.20	13.13 ± 0.18
	*p* value	<0.001	<0.001	<0.001	<0.001	<0.001	0.038	0.010	0.003	0.002	0.115	0.522	0.305	0.349
	General	8.64 ± 0.18	9.06 ± 0.19	9.16 ± 0.18	9.23 ± 0.17	9.30 ± 0.16	9.67 ± 0.15	10.11 ± 0.16	11.12 ± 0.17	11.64 ± 0.17	12.44 ± 0.17	12.88 ± 0.17	12.99 ± 0.17	13.18 ± 0.15
Tail Length	Male	22.61 ± 0.18	23.84 ± 0.15	24.65 ± 0.19	26.72 ± 0.19	29.57 ± 0.19	32.79 ± 0.20	35.91 ± 0.23	38.79 ± 0.22	41.17 ± 0.20	43.01 ± 0.21	45.14 ± 0.21	46.01 ± 0.22	46.47 ± 0.23
	Female	21.10 ± 0.21	22.40 ± 0.22	23.10 ± 0.22	24.25 ± 0.20	26.30 ± 0.39	29.10 ± 0.37	32.10 ± 0.31	35.05 ± 0.29	38.00 ± 0.31	40.55 ± 0.28	42.85 ± 0.47	44.00 ± 0.43	45.25 ± 0.32
	*p* value	<0.001	<0.001	<0.001	<0.001	<0.001	<0.001	<0.001	<0.001	<0.001	<0.001	<0.001	<0.001	0.023
	General	22.35 ± 0.17	23.58 ± 0.15	24.38 ± 0.18	26.28 ± 0.20	29.00 ± 0.24	32.14 ± 0.26	35.25 ± 0.28	38.14 ± 0.26	40.61 ± 0.23	42.57 ± 0.22	44.74 ± 0.22	45.65 ± 0.22	46.26 ± 0.20
Traits	Sex	45 days	60 days	75 days	3 months	4 months	5 months	6 months	7 months	8 months	9 months	10 months	11 months	12 months
Muzzle Circumference	Male	18.47 ± 0.20	18.94 ± 0.20	19.01 ± 0.19	20.30 ± 0.19	21.52 ± 0.19	23.47 ± 0.18	25.81 ± 0.19	27.43 ± 0.17	28.35 ± 0.15	28.89 ± 0.15	29.69 ± 0.14	30.41 ± 0.12	31.13 ± 0.11
	Female	17.90 ± 0.42	18.60 ± 0.30	19.05 ± 0.26	19.65 ± 0.27	20.65 ± 0.42	21.70 ± 0.42	23.40 ± 0.37	24.90 ± 0.41	26.45 ± 0.53	27.05 ± 0.54	27.50 ± 0.51	27.90 ± 0.55	28.20 ± 0.55
	*p* value	0.219	0.344	0.905	0.060	0.062	<0.001	<0.001	<0.001	0.006	0.008	<0.001	0.001	<0.001
	General	18.37 ± 0.18	18.88 ± 0.17	19.01 ± 0.16	20.19 ± 0.17	21.36 ± 0.18	23.16 ± 0.19	25.39 ± 0.21	26.99 ± 0.20	28.01 ± 0.18	28.57 ± 0.18	29.30 ± 0.18	29.97 ± 0.19	30.62 ± 0.20
Body Length	Male	35.40 ± 0.20	37.78 ± 0.23	39.02 ± 0.26	42.48 ± 0.24	46.65 ± 0.16	49.78 ± 0.18	53.79 ± 0.19	58.22 ± 0.15	63.81 ± 0.19	65.94 ± 0.20	68.41 ± 0.21	70.18 ± 0.22	72.85 ± 0.24
	Female	33.45 ± 0.50	35.50 ± 0.42	36.65 ± 0.17	39.50 ± 0.13	42.80 ± 0.25	46.65 ± 0.40	50.50 ± 0.33	55.80 ± 0.29	59.35 ± 0.33	62.80 ± 0.20	65.25 ± 0.34	67.35 ± 0.26	68.25 ± 0.21
	*p* value	<0.001	<0.001	<0.001	<0.001	<0.001	<0.001	<0.001	<0.001	<0.001	<0.001	<0.001	<0.001	<0.001
	General	35.06 ± 0.21	37.38 ± 0.23	38.60 ± 0.25	41.96 ± 0.25	45.98 ± 0.24	49.23 ± 0.23	53.21 ± 0.24	57.79 ± 0.18	63.03 ± 0.28	65.39 ± 0.23	67.85 ± 0.25	69.68 ± 0.23	72.04 ± 0.31
Height at Withers	Male	24.60 ± 0.12	29.14 ± 0.26	34.01 ± 0.30	39.97 ± 0.22	43.07 ± 0.20	46.96 ± 0.21	51.17 ± 0.20	55.98 ± 0.20	59.82 ± 0.27	64.48 ± 0.30	66.90 ± 0.23	68.46 ± 0.22	70.07 ± 0.22
	Female	22.70 ± 0.21	26.30 ± 0.17	31.00 ± 0.22	36.35 ± 0.24	39.85 ± 0.35	42.60 ± 0.27	46.30 ± 0.24	52.40 ± 0.10	57.05 ± 0.16	60.30 ± 0.31	63.15 ± 0.46	64.75 ± 0.52	65.90 ± 0.49
	*p* value	<0.001	<0.001	<0.001	<0.001	<0.001	<0.001	<0.001	<0.001	<0.001	<0.001	<0.001	<0.001	<0.001
	General	24.27 ± 0.14	28.64 ± 0.26	33.48 ± 0.28	39.34 ± 0.25	42.50 ± 0.24	46.20 ± 0.28	50.31 ± 0.30	55.35 ± 0.25	59.34 ± 0.27	63.75 ± 0.33	66.24 ± 0.28	67.81 ± 0.27	69.34 ± 0.29
Chest Depth	Male	12.55 ± 0.17	13.24 ± 0.16	13.76 ± 0.17	14.55 ± 0.17	15.53 ± 0.16	16.82 ± 0.17	18.91 ± 0.17	21.00 ± 0.17	22.59 ± 0.18	23.72 ± 0.18	24.77 ± 0.21	25.87 ± 0.20	26.69 ± 0.19
	Female	12.05 ± 0.17	12.50 ± 0.18	13.10 ± 0.15	13.60 ± 0.15	14.75 ± 0.19	15.80 ± 0.25	17.30 ± 0.34	19.45 ± 0.26	21.05 ± 0.34	22.20 ± 0.27	23.00 ± 0.30	23.70 ± 0.26	24.25 ± 0.26
	*p* value	0.045	0.005	0.006	<0.001	0.004	0.007	<0.001	<0.001	<0.001	<0.001	<0.001	<0.001	<0.001
	General	12.46 ± 0.14	13.10 ± 0.14	13.64 ± 0.15	14.37 ± 0.15	15.38 ± 0.14	16.63 ± 0.15	18.61 ± 0.17	20.70 ± 0.17	22.30 ± 0.18	23.43 ± 0.18	24.44 ± 0.21	25.46 ± 0.21	26.23 ± 0.21
Chest Width	Male	8.01 ± 0.19	8.94 ± 0.19	9.89 ± 0.20	10.51 ± 0.19	11.51 ± 0.20	12.56 ± 0.19	15.24 ± 0.19	17.02 ± 0.19	18.48 ± 0.19	18.98 ± 0.19	19.51 ± 0.19	20.03 ± 0.19	20.64 ± 0.18
	Female	7.95 ± 0.23	8.75 ± 0.17	9.05 ± 0.09	9.20 ± 0.08	9.80 ± 0.08	11.30 ± 0.13	12.95 ± 0.26	15.25 ± 0.23	16.50 ± 0.15	17.55 ± 0.14	18.25 ± 0.15	18.75 ± 0.13	19.30 ± 0.15
	*p* value	0.065	0.054	<0.001	<0.001	<0.001	<0.001	<0.001	<0.001	<0.001	<0.001	<0.001	<0.001	<0.001
	General	8.00 ± 0.16	8.91 ± 0.16	9.74 ± 0.17	10.28 ± 0.17	11.21 ± 0.18	12.34 ± 0.17	14.84 ± 0.20	16.71 ± 0.18	18.14 ± 0.19	18.73 ± 0.17	19.28 ± 0.17	19.80 ± 0.17	20.41 ± 0.17
Height at Rump	Male	24.24 ± 0.12	28.67 ± 0.30	33.41 ± 0.35	39.18 ± 0.22	42.30 ± 0.21	45.73 ± 0.21	50.77 ± 0.22	55.59 ± 0.22	59.02 ± 0.23	63.73 ± 0.22	66.67 ± 0.24	67.94 ± 0.21	69.05 ± 0.21
	Female	23.35 ± 0.92	25.95 ± 0.16	30.05 ± 0.18	36.74 ± 0.20	39.80 ± 0.30	42.85 ± 0.17	47.00 ± 0.15	52.50 ± 0.13	56.50 ± 0.28	60.40 ± 0.45	62.60 ± 0.63	65.15 ± 0.72	66.40 ± 0.74
	*p* value	0.360	<0.001	<0.001	<0.001	<0.001	<0.001	<0.001	<0.001	<0.001	<0.001	<0.001	<0.001	<0.001
	General	24.08 ± 0.19	28.19 ± 0.29	32.82 ± 0.33	38.75 ± 0.22	41.86 ± 0.22	45.22 ± 0.23	50.11 ± 0.26	55.05 ± 0.24	58.57 ± 0.24	63.14 ± 0.26	65.95 ± 0.31	67.45 ± 0.25	68.58 ± 0.25
Traits	Sex	45 days	60 days	75 days	3 months	4 months	5 months	6 months	7 months	8 months	9 months	10 months	11 months	12 months
Rump Depth	Male	8.34 ± 0.14	9.00 ± 0.15	9.67 ± 0.17	10.59 ± 0.17	11.88 ± 0.18	13.36 ± 0.17	14.73 ± 0.17	17.22 ± 0.20	18.58 ± 0.19	19.46 ± 0.18	20.06 ± 0.17	20.38 ± 0.15	20.85 ± 0.17
	Female	7.80 ± 0.30	8.35 ± 0.34	8.65 ± 0.37	9.45 ± 0.38	10.35 ± 0.43	11.85 ± 0.51	12.90 ± 0.62	15.65 ± 0.45	18.05 ± 0.19	18.90 ± 0.22	19.75 ± 0.20	20.35 ± 0.15	20.80 ± 0.17
	*p* value	0.113	0.073	0.014	0.008	0.001	0.001	0.002	0.002	0.209	0.158	0.414	0.924	0.89
	General	8.24 ± 0.12	8.88 ± 0.13	9.49 ± 0.16	10.39 ± 0.16	11.61 ± 0.18	13.09 ± 0.18	14.41 ± 0.19	16.94 ± 0.19	18.49 ± 0.16	19.36 ± 0.15	20.00 ± 0.14	20.37 ± 0.13	20.84 ± 0.14
Rump Width	Male	8.34 ± 0.15	9.14 ± 0.18	9.77 ± 0.19	10.81 ± 0.20	12.36 ± 0.21	14.29 ± 0.21	15.60 ± 0.20	18.88 ± 0.20	19.93 ± 0.19	20.90 ± 0.20	21.37 ± 0.21	21.57 ± 0.20	22.07 ± 0.20
	Female	7.20 ± 0.15	7.65 ± 0.15	8.15 ± 0.15	8.65 ± 0.15	9.20 ± 0.13	10.30 ± 0.11	11.40 ± 0.10	13.60 ± 0.19	14.75 ± 0.08	15.95 ± 0.14	16.80 ± 0.15	17.25 ± 0.13	17.90 ± 0.10
	*p* value	<0.001	<0.001	<0.001	<0.001	<0.001	<0.001	<0.001	<0.001	<0.001	<0.001	<0.001	<0.001	<0.001
	General	8.14 ± 0.14	8.88 ± 0.17	9.49 ± 0.18	10.43 ± 0.20	11.80 ± 0.24	13.59 ± 0.27	14.86 ± 0.27	17.95 ± 0.32	19.02 ± 0.31	20.03 ± 0.30	20.57 ± 0.29	20.81 ± 0.28	21.34 ± 0.27
Ridge Height	Male	23.62 ± 0.13	28.11 ± 0.30	33.01 ± 0.37	37.71 ± 0.24	41.22 ± 0.22	44.72 ± 0.22	49.23 ± 0.22	54.62 ± 0.26	57.65 ± 0.26	62.62 ± 0.26	65.54 ± 0.25	65.57 ± 1.30	68.04 ± 0.22
	Female	22.85 ± 0.92	25.50 ± 0.13	29.35 ± 0.21	35.35 ± 0.38	38.90 ± 0.40	41.80 ± 0.34	46.35 ± 0.27	51.80 ± 0.25	55.20 ± 0.27	58.00 ± 0.24	61.15 ± 0.48	62.75 ± 0.55	64.15 ± 0.68
	*p* value	0.424	<0.001	<0.001	<0.001	<0.001	<0.001	<0.001	<0.001	<0.001	<0.001	<0.001	<0.001	<0.001
	General	23.49 ± 0.19	27.65 ± 0.28	32.36 ± 0.36	37.29 ± 0.24	40.81 ± 0.22	44.21 ± 0.24	48.72 ± 0.24	54.13 ± 0.26	57.22 ± 0.26	61.81 ± 0.32	64.77 ± 0.31	65.07 ± 1.08	67.35 ± 0.29
Neck Coat Length	Male	3.01 ± 0.06	3.40 ± 0.06	3.62 ± 0.07	4.15 ± 0.07	4.97 ± 0.08	5.65 ± 0.07	6.75 ± 0.08	7.62 ± 0.09	8.54 ± 0.10	9.27 ± 0.09	9.85 ± 0.09	10.43 ± 0.09	11.00 ± 0.09
	Female	3.05 ± 0.14	3.45 ± 0.12	3.75 ± 0.11	4.20 ± 0.08	4.85 ± 0.13	5.60 ± 0.18	6.80 ± 0.15	7.70 ± 0.13	8.65 ± 0.17	9.25 ± 0.15	9.60 ± 0.18	10.20 ± 0.21	10.75 ± 0.19
	*p* value	0.797	0.747	0.433	0.798	0.475	0.74	0.813	0.734	0.645	0.900	0.247	0.287	0.252
	General	3.01 ± 0.06	3.41 ± 0.05	3.64 ± 0.06	4.16 ± 0.06	4.95 ± 0.07	5.64 ± 0.07	6.76 ± 0.07	7.64 ± 0.08	8.56 ± 0.09	9.27 ± 0.07	9.80 ± 0.07	10.39 ± 0.07	10.95 ± 0.07

At 12 months of age, the average head length, face length, head width, head circumference and muzzle circumference of Kars Shepherd dogs were found 27.06, 10.64, 13.34, 51.92 and 30.62 cm, respectively. In all age groups, the differences between the sex groups for head length were non‐significant (*p* > 0.05) between the 5th and 9th month of age; however, significant (*p* < 0.05) at other ages. Differences in face length between the sex groups were significant only at 45 days of age (*p* < 0.05). Differences between sex groups in terms of head width, head circumference and ear width means were significant for all age groups (*p* < 0.05). No statistically significant differences (*p* > 0.05) in muzzle circumference between males and females were observed between 45 days and 4 months of age. However, from 5 months to 12 months of age, males demonstrated significantly larger muzzle circumferences than females (*p* < 0.05). Mean ear length, ear width and distance between the two ears of Kars Shepherd dog puppies at the age of 12 months were 16.79, 12.12 and 12.94 cm, respectively. Sex‐related differences in mean ear length were not significant between 45 days and 5 months of age (*p* > 0.05), however were significantly higher in males at other ages than in females (*p* < 0.05). All sex‐related differences in ear width according to age were statistically significant (*p* < 0.05). Differences between sex groups in terms of distance between the two ears according to age were statistically insignificant at 45 days of age (*p* > 0.05), but significant at all other ages (*p* < 0.05).

Mean values of chest circumference, leg length, front wrist circumference, rear wrist circumference and tail length of Kars Shepherd dog puppies at the age of 12 months were found 79.77, 42.52, 13.70, 13.18 and 46.26 cm, respectively. Difference between sex groups in mean chest circumference was statistically significant at all ages (*p* < 0.05). Differences between sex groups in terms of mean leg length were statistically non‐significant at 45 days of age (*p* > 0.05), but statistically significant at all other ages (*p* < 0.05). Statistically significant (*p* < 0.05) sex‐based variations in the front wrist circumference were observed between the ages of 45 days and 8 months. However, these differences were not statistically significant (*p* > 0.05) in older age groups. The differences between the means of rear wrist circumference among sex group were statistically significant between 45 days and 8 months of age (*p *< 0.05) but remained non‐significant at other ages (*p *> 0.05). Differences in tail length between sex groups were statistically significant (*p* < 0.05) in all age groups.

The mean body length, height at withers, chest depth, chest width, height at rump, rump depth, rump width, ridge height and neck coat length of Kars Shepherd dog puppies at the age of 12 months were 72.04, 69.34, 26.23, 20.41, 68.58, 20.84, 21.34, 67.35 and 10.95 cm, respectively. Differences between sex groups in terms of body length and height at withers were statistically significant at all ages (*p* < 0.001). Similarly, differences between the sex groups in terms of chest depth were statistically significant at all ages (*p* < 0.05). The differences in the chest width between sex groups were statistically non‐significant between 45 and 60 days of age (*p* > 0.05), but statistically significant at other ages (*p* < 0.001). Sex‐related differences in height at rump were non‐significant at 45 days of age (*p* > 0.05) but were significant for other ages (*p* < 0.001). Differences in rump depth were statistically significant (*p* < 0.05) between 75th days and 7 months of age but remained non‐significant for other ages (*p* > 0.05). The rump width demonstrated statistically significant sex‐based differences across all the age groups (*p* < 0.001). At 45 days of age, differences in ridge height were not statistically significant (*p* > 0.05); however, they became significant in the subsequent age groups (*p* < 0.001). No statistically significant sex‐related variation was observed in the neck coat length at any age (*p* > 0.05).

### 3.5 Physiological Characteristics of New‐Born Puppies

**FIGURE 1:**
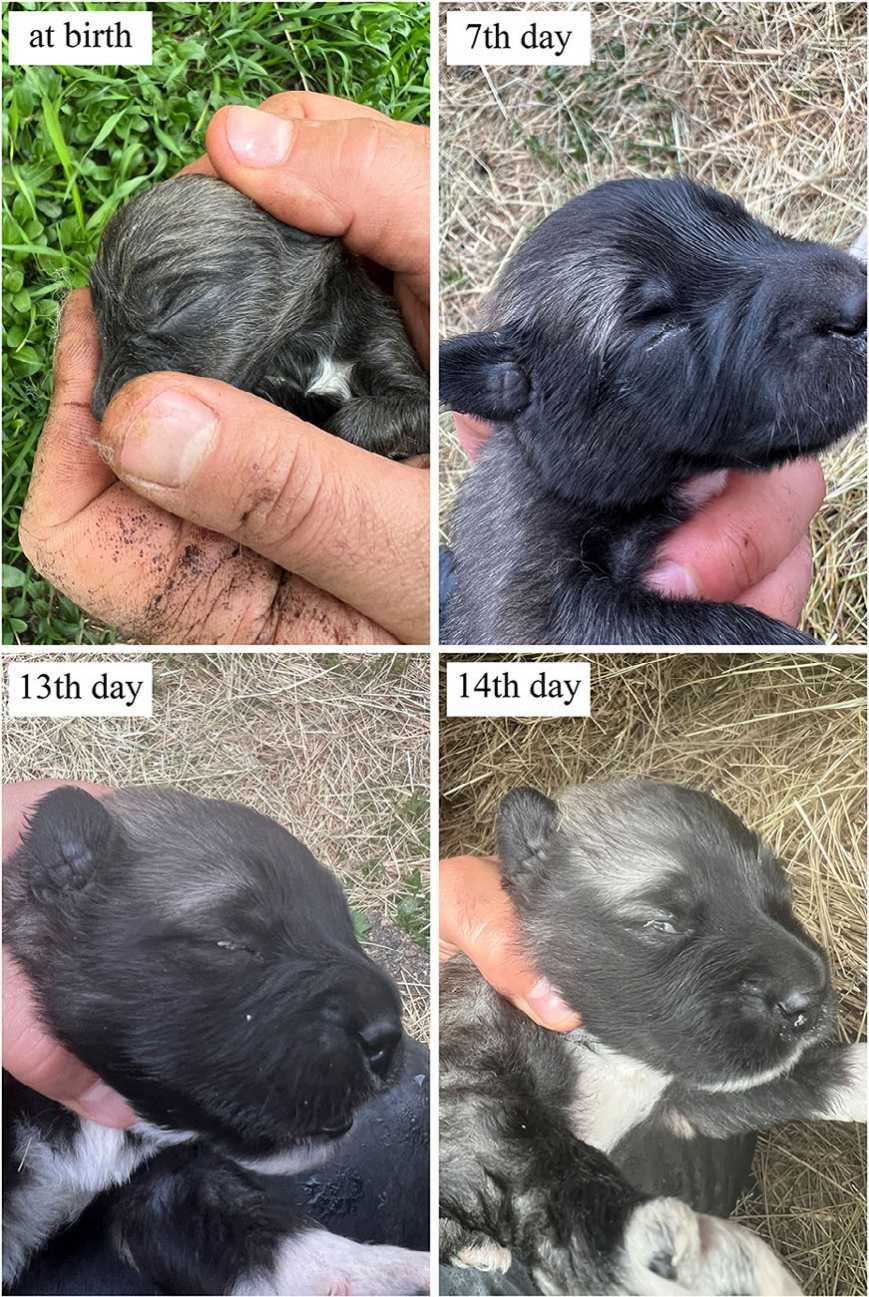
The appearance of the eyelid of a Kars Shepherd Dog puppy at different ages and the opening and activation of the eyelid on the average 14th day.

**FIGURE 2:**
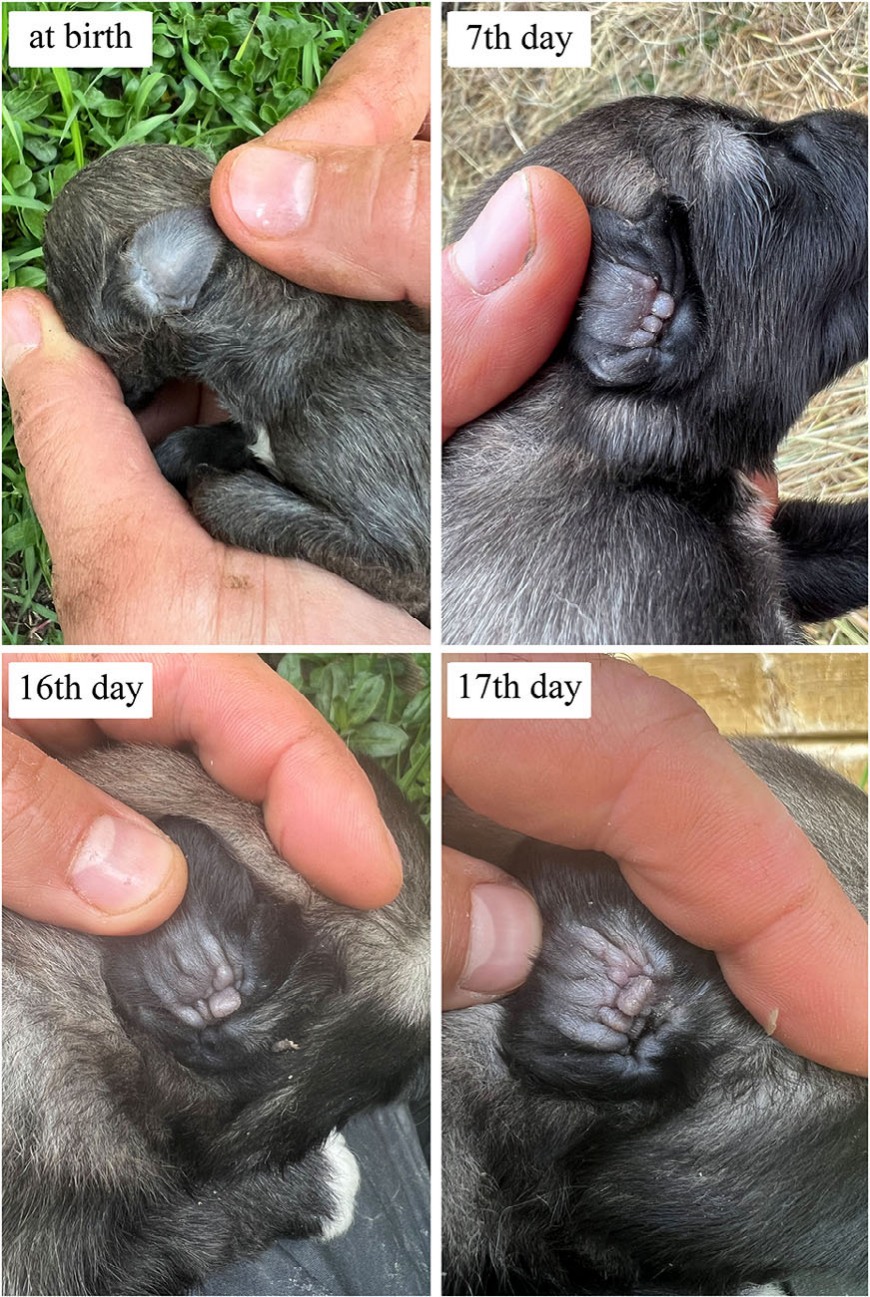
The appearance of the external ear canal of a Kars Shepherd Dog puppy at different ages and the opening of the external ear canal on the average 17th day.

**FIGURE 3:**
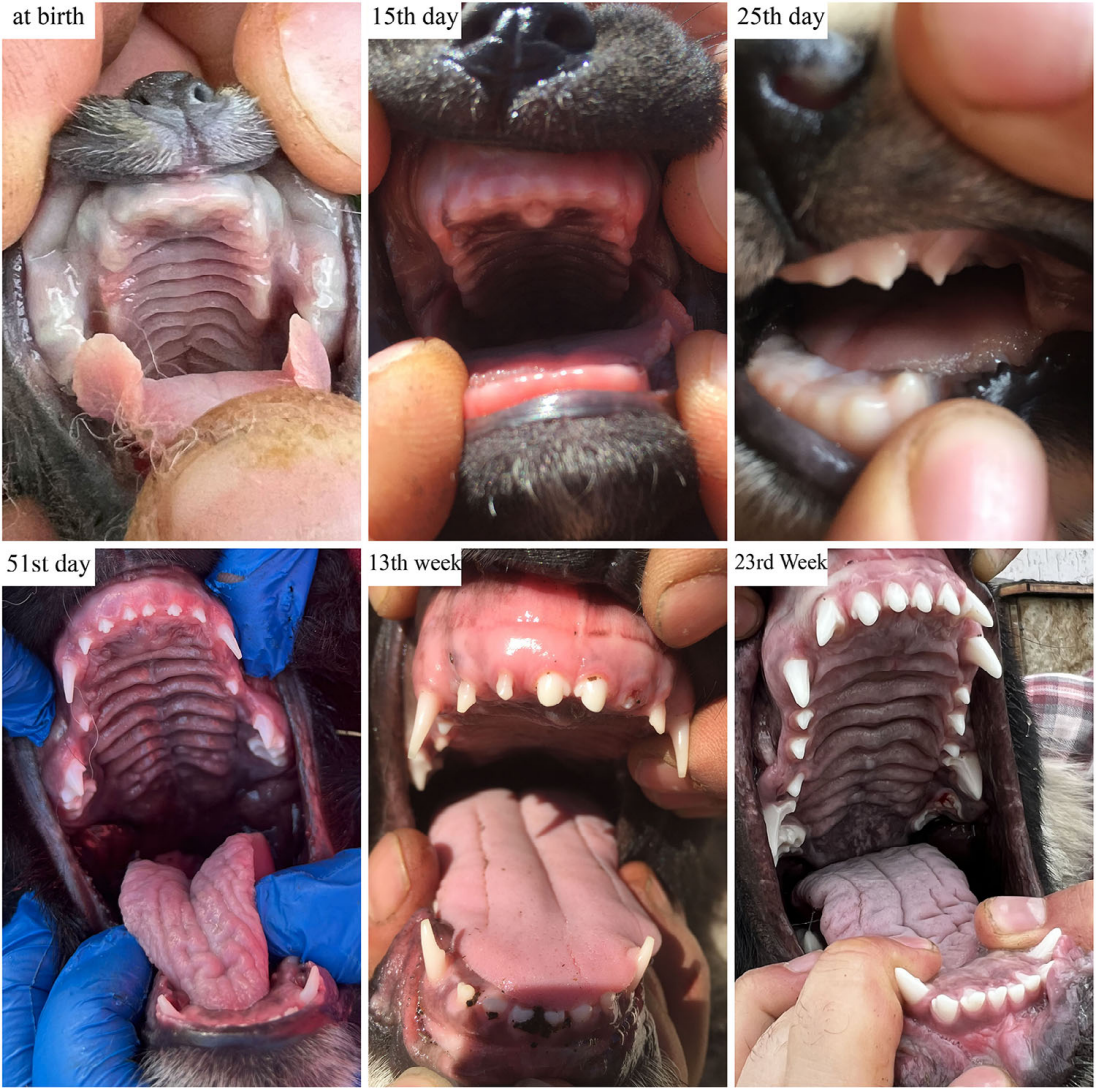
View of deciduous and permanent teeth of a same Kars Shepherd puppy from birth. At birth: Appearance of the toothless mouth of a newborn puppy, 15th day: Puppy's toothless mouth 14 days after birth, 25th day: Age at the first eruption of the puppy's deciduous teeth, 51th day: The age at which puppy's deciduous teeth are complete, 13th week: Age at the first eruption of the puppy's permanent teeth, 23rd week: The age at which puppy's permanent teeth are complete.

**FIGURE 4:**
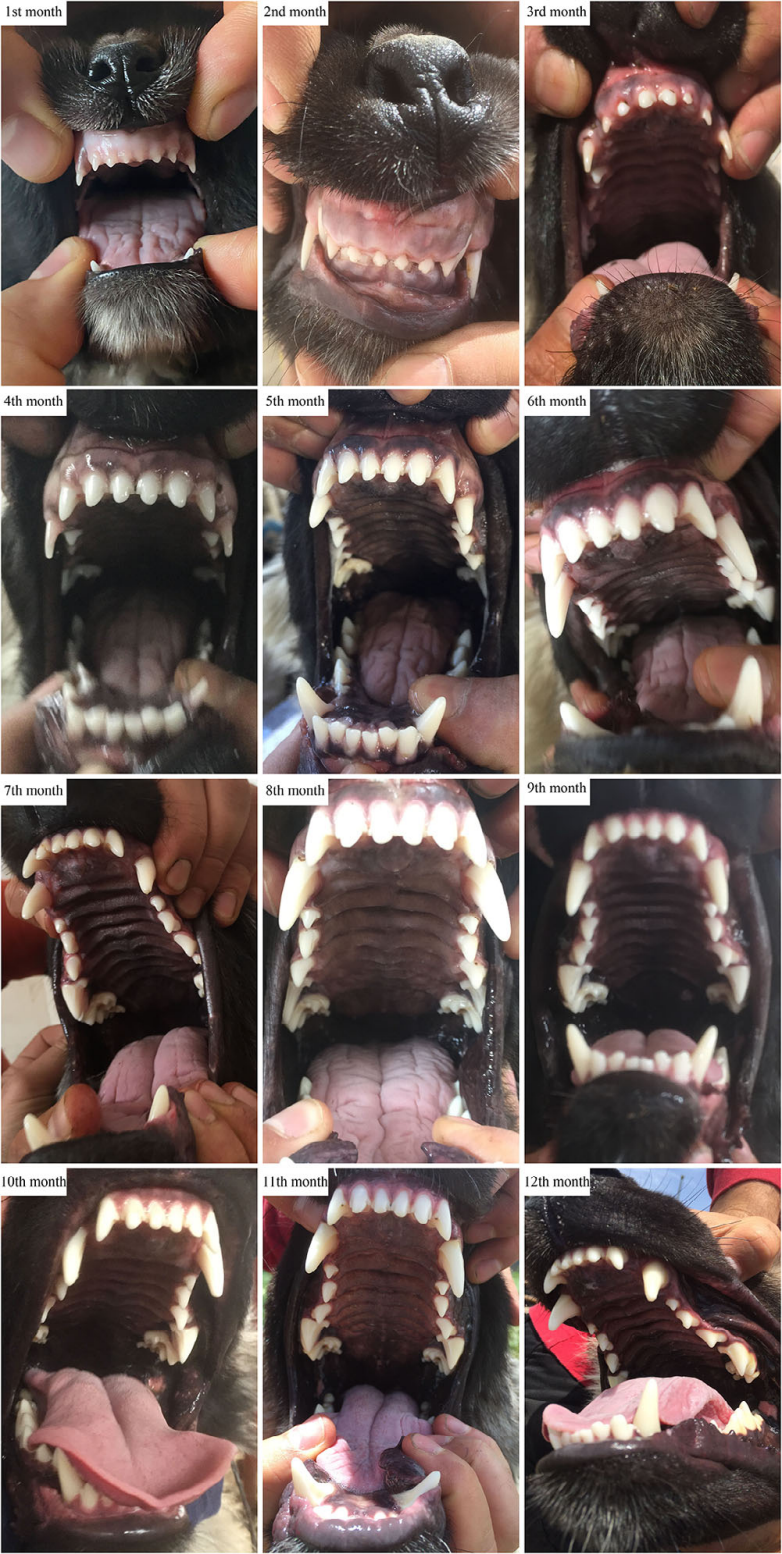
Appearance of the teeth of a same Kars Shepherd puppy from 1 month to 12 months of age.

The mean of ages at which the new‐born Kars Shepherd dog puppies' eyelid first opened, the external ear canal first opened, the first deciduous teeth erupted, the deciduous teeth were completed, the first permanent teeth erupted and the permanent teeth completed were determined to be 14.02, 16.95, 25.21, 51.56 days, 12.51 and 22.72 weeks, respectively (Figure 1, 2, 3). The appearance of the teeth of the Kars Shepherd dog puppy from 1 to 12 months of age is illustrated in Figure 4. The age at which the external ear canal first opened was observed to be 17.40 days in females and 16.85 days in males, and the differences between the sex groups were found to be statistically significant (*p* < 0.05) (Table 6).

**TABLE 6:** Mean ages of newborn Kars Shepherd dog puppies at first opening of the eyelids, external ear canal opened, and deciduous and permanent teeth erupted.

Feature	Gender	*n*	X̄ ± Sx̄	Min.	Max.	*p* value
Age at first opening of the eyelids (days)	Female	10	14.10 ± 0.23	13	15	0.694
	Male	47	14.00 ± 0.10	13	15	
	General	57	14.02 ± 0.09	13	15	
Age at first opening of the external ear canal (days)	Female	10	17.40 ± 0.22^b^	16	18	0.021
	Male	47	16.85 ± 0.09^a^	16	18	
	General	57	16.95 ± 0.09	16	18	
Age at first eruption of deciduous teeth (days)	Female	10	25.00 ± 0.55	22	27	0.779
	Male	47	25.26 ± 0.22	21	27	
	General	57	25.21 ± 0.20	21	27	
Age at complete emergence of deciduous teeth (days)	Female	10	51.80 ± 0.90	48	57	0.700
	Male	47	51.51 ± 0.28	48	58	
	General	57	51.56 ± 0.28	48	58	
Age at first eruption of permanent teeth (weeks)	Female	10	12.70 ± 0.15	12	13	0.194
	Male	47	12.47 ± 0.07	12	13	
	General	57	12.51 ± 0.06	12	13	
Age at complete emergence of permanent teeth (weeks)	Female	10	22.60 ± 0.16	22	23	0.508
	Male	47	22.74 ± 0.09	22	24	
	General	57	22.72 ± 0.08	22	24	

^a,b^ Different letters in the same column indicate statistical differences.

### 3.6 Morphological Characteristics of Kars Shepherd Dogs

**FIGURE 5:**
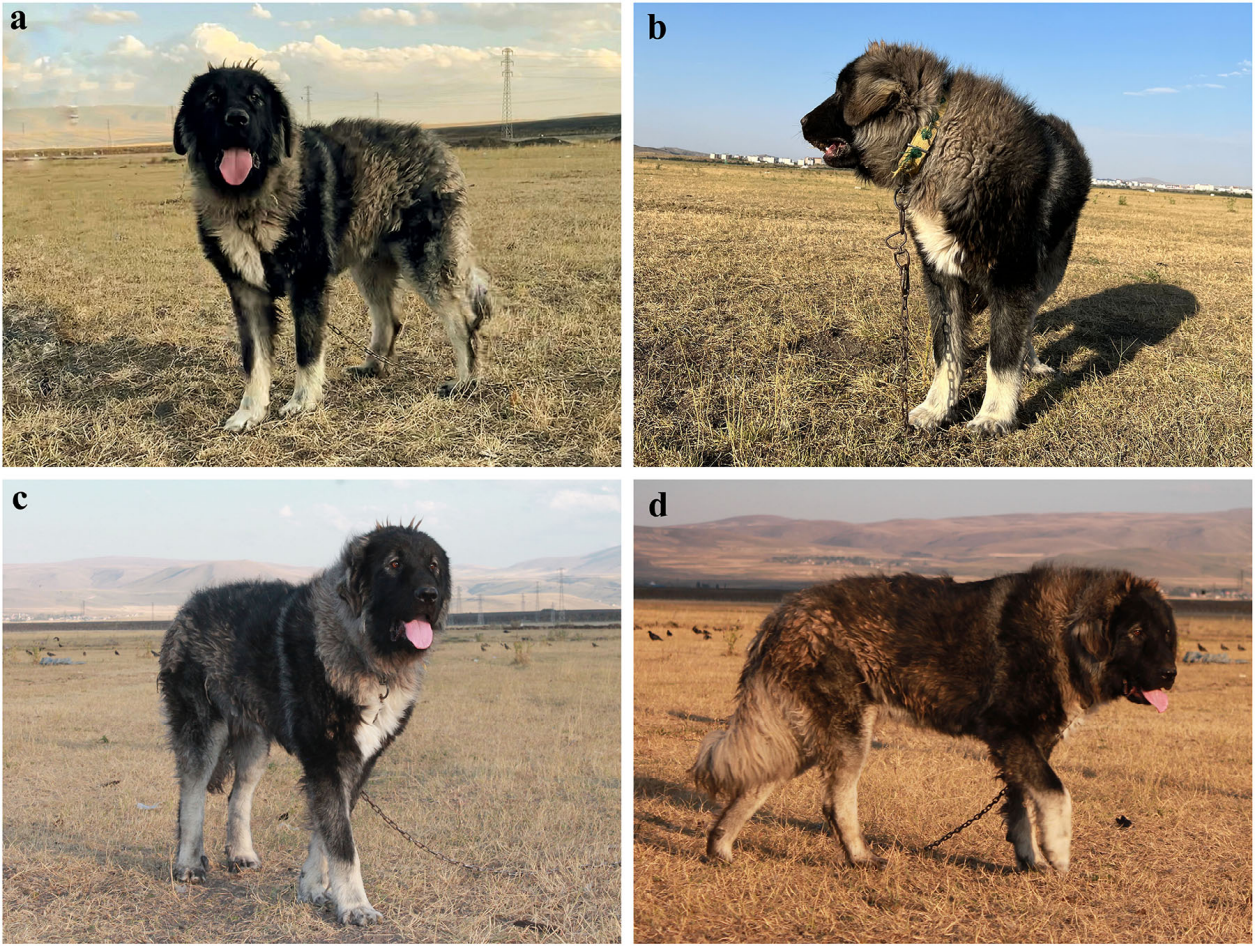
Photographs of a 12‐month‐old Kars Shepherd dog from various angles presenting body structure and appearance (a–d).

**FIGURE 6:**
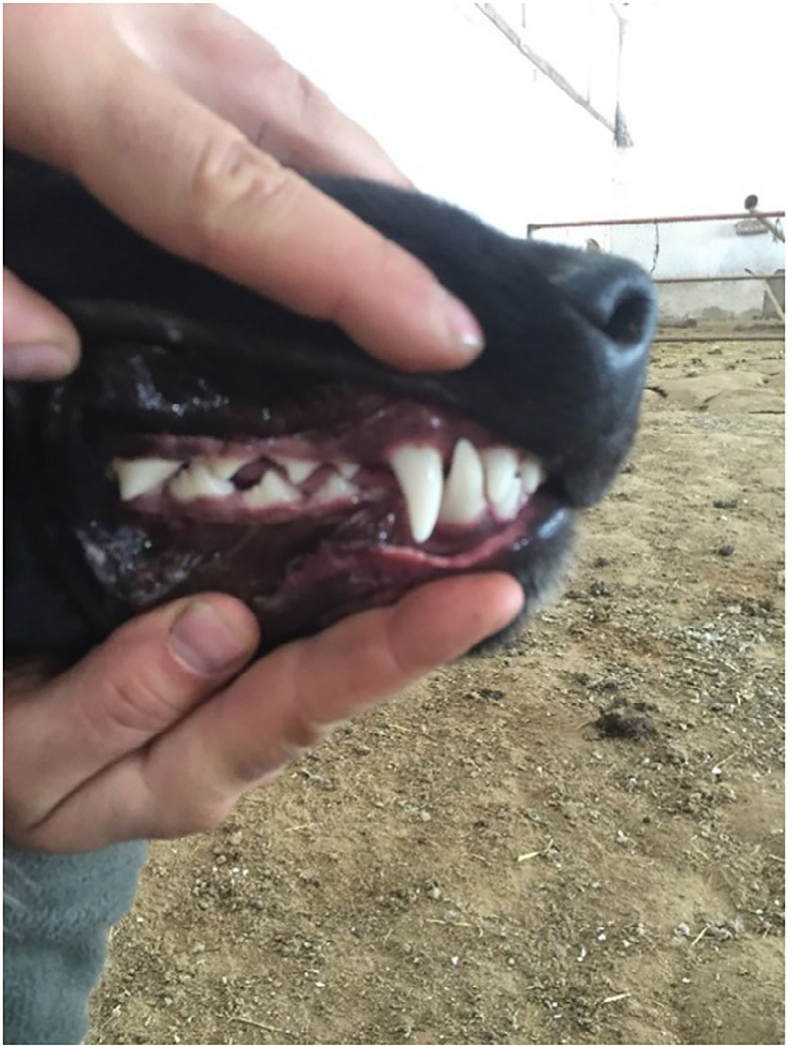
Scissors bite formation by closing of the teeth and jaw of the Kars Shepherd dog.

Kars Shepherd dogs are large, muscular and possess a robust bone structure. From the frontal view, they presented a strikingly majestic appearance due to their large head and broad chests. The average withers and rump height were closely aligned. When viewed laterally, the backline was parallel to the ground, and the body exhibited a slightly rectangular shape (Figure 5d). A distinctive feature of these dogs was the black facial mask, which typically covered the muzzle, nose and eyes. The forehead was broad with a pronounced stop (the area between the orbits, os frontale and os nasale) (Figure 5a). The eyes were medium‐sized, round and brown or shades of brown. Their expression was stern, serious and attentive. The eyelids were black, and the area around the eyes was surrounded by lighter‐coloured fur, creating a characteristic light‐coloured frame (Figure 5c). The ears of Kars Shepherd dogs were broad, medium‐length, drooping and triangular. The ear colour was generally black or grey‐toned, with the tone darkening towards the tips (apex) (Figure 5b). These dogs had a large muzzle and a broad, blunt nose bridge. The upper lines of the muzzle and skull were parallel. The lips were thick, black and non‐drooping (Figure 5b). The teeth were strong, bright white and met in a scissors bite when the jaws closed (Figure 6). The chest was broad, deep and muscular, often featuring localized white fur on the anterior chest surface (Figure 5a). The legs were strong and muscular, with longer fur on the rear side of the forelimbs and hind limbs, creating a fringe‐like appearance. The coat colour on the legs lightened from proximal to distal (Figure 5c). The tail was covered with dense and long fur, which may lighten to grey or white tones at the tip. When relaxed, the tail hung toward the stifle, and while in alert condition, it arched over the back in a crescent shape (Figure 5d). The Kars Shepherd dogs coat was generally long; however, medium‐ and short‐haired variations were also present. The coat was typically grey and black, while the fur covering the neck was lighter than the rest of the body colour (Figure 5b).

## 4 Discussion and Conclusion

This study is the first study evaluate the Kars Shepherd dogs' growth performance and reproductive characteristics. Therefore, the findings of the present study were first compared with other Turkish Shepherd dogs and then with other Shepherd dogs breeds worldwide.

In this study, estrus in Kars Shepherd dogs was observed year‐round. These findings are consistent with those reported by other studies of Jöchle and Andersen (1977), Forsberg and Persson (2007) and Tepeli and Çetin (2000). The mean inter‐estrus interval was 234.72 days, which is within the average inter‐estrus interval (5–12 months) reported for dogs (Concannon 2011; Erol and Atasoy 2010). This comparatively inter‐estrus interval in dogs is advantageous for breeders, as it results in more litter per year. The mean duration of proestrus and estrus in Kars Shepherd dogs were 7.12 and 6.42 days, respectively. These values are consistent with previous studies that reported proestrus and estrus durations of 3–17 and 3–21 days, respectively (Erol 2008; Freshman 1991).

The mean age at pubertal estrus in Kars Shepherd dogs was 427.0 days. This mean is similar to the 423.0 days reported for Kangal Shepherd dogs by Kirmizi (1991). However, this age is higher than the age reported (408.5–411.8 days) for Kangal Shepherd dogs (Altıner 1998; Erol and Atasoy 2010; Tepeli and Çetin 2000), and lower than the 21.86 and 15.46 months old reported for German Shepherd and Labrador Retrievers, respectively (Sevimli 2007). It is thought that a lower age at pubertal estrus is favourable for breeders because it enables breeding at an early age. The average mating duration in the present study was recorded at 18.98 min. This duration is similar to the average reported values of 19–21 min for other Shepherd dogs (Gönül 1996; Tepeli and Çetin 2000). The estrus rate was 100% in Kars Shepherd dogs, which is higher than the average range (70%–94%) reported for other Shepherd dog breeds (Kirmizi 1991; Mutembei et al. 2000; Tepeli and Çetin 2000), and similar to the 100% rate found for the Kangal Shepherd dog (Erol and Atasoy 2010).

The average gestation period in this study was found to be 62.78 days, which was consistent with the reported average gestation period of 63 days in dogs (Alaçam 1999; Erol and Atasoy 2010; Zonturlu et al. 2010). The average litter size of 5.03 puppies per birth observed in this study was similar to the litter size of 5.09 reported by Erol (2008) for Kangal Shepherd dogs. Similarly, another study reported an average litter size of 5.4 across 224 dog breeds (Borge et al. 2011). However, the litter size obtained in this study was lower than the 6.2–8.9 reported for Kangal Shepherd dogs in other studies (Altıner 1998; Ograk 2009; Tepeli and Çetin 2000). In this study, the stillbirth rate was 27.95%, similar to the rates of 26.7% and 37.2% reported for St. Bernard and Bernese Mountain dog breeds, respectively (Nielen et al. 1998). While the stillbirth rate was higher than the 1.32–13.66% reported for Kangal Shepherd dogs (Erol and Atasoy 2010; Tepeli and Çetin 2000).

The survival rates at 45 days (weaning age) and 2 months old of Kars dogs puppies were 87.1% and 84.5%, respectively. These rates closely align with the 88.8% and 86.8%–87.5% reported for Kangal Shepherd dog puppies at these ages (Erol 2008; Tepeli and Çetin 2000).

In this study, the mean birth weight of Kars Shepherd dog puppies was determined to be 510.96 g. This value was found to be analogous to the values reported for Turkish Shepherd dogs (Tepeli and Çetin 2000; Tepeli et al. 2003; Erol and Atasoy 2010; Yildirim et al. 2012), which ranged from 519 to 543 g.

In this study, the mean live weight of 12‐month‐old Kars Shepherd dogs was 40.63 kg, which was higher than the 33.32 kg reported for the same genotype (Kirmizibayrak 2004). In some studies, similar to this one, live weights of 36.89–45.62 kg were reported in Turkish Shepherd dogs (Altıner 1998; Atasoy et al. 2011; Özbeyaz 1994; Tepeli et al. 2003). The higher live weight determined in this study compared to the same genotype may be a result of improved management and feeding practices.

The average head length, head circumference and ear length of 12‐month‐old Kars Shepherd Dogs were 27.06, 51.92 and 16.79 cm, respectively. The head length was lower than the 29.5–30.8 cm reported for the same breed (Erdoğan et al. 2012; Kirmizibayrak 2004; Tepeli et al. 2017). However, it was similar to the 27.8–28.4 cm reported for other Turkish shepherd dogs (Atasoy et al. 2011; Tepeli and Çetin 2000; Yertürk and Bozkaya 2012). The average head circumference observed in this study was similar to the results of another study (52.38 cm) on the same genotype (Kirmizibayrak 2004) and within the range of 46.99–55.7 cm reported for other Turkish Shepherd dogs (Ograk et al. 2018; Tepeli et al. 2003; Tepeli and Çetin 2000). The ear length in this study was found higher than the 12.2–13.2 cm reported for the Kangal Shepherd dog (Özbeyaz 1994; Tepeli and Çetin 2000), 12.09–13.48 cm found for the German Shepherd dog, Labrador Retriever and Mallinois (Dirlik 2008). Differences in the results can be attributed to the age at measurement and genetic variation among breeds.

The average height at withers, height at rump, rump width and body length of 12‐month‐old Kars Shepherd dogs were 69.34, 68.58, 21.34 and 72.04 cm, respectively. These values were higher than the averages of 62.65, 61.45, 19.70 and 65.35 cm reported for the same genotype by Kirmizibayrak (2004). On the contrary, presented results were lower than the values reported for larger breeds such as Malakli and Bozova dogs, with height at withers 76.60–77.0 cm, height at rump 77.50–77.20 cm and body length 78.50–81.75 cm (Atasoy et al. 2014; Yertürk and Bozkaya 2012). In addition, the values found in this study were similar to the values of height at withers (68.93 cm), height at rump (70.44 cm) and rump width (19.14 cm) reported for Kangal Shepherd dogs (Tepeli 1996), but different from body length (63.76 cm).

The average chest circumference, depth and width were 79.77, 26.23 and 20.41 cm, respectively. These values are similar to the chest depth (25.29 cm), and higher than those reported for chest circumference (74.73 cm) and chest width (16.9 cm) for the same genotype (Kırmızıbayrak 2004). They are also similar to the chest circumference (80.71 cm), chest width (20.16 cm) and chest depth (27.61 cm) reported for Kangal Shepherd dog.

In conclusion, this study has, for the first time, scientifically determined many characteristics of the Kars Shepherd dog, which is a significant animal genetic resource of Türkiye. These include significant morphological characteristics, fertility traits, puppy growth characteristics, body measurements for age, information on tooth changes in puppies and the age at which hearing and vision become active. Notably, some of these traits (some morphological traits, body measurements according to age in puppies, tooth change information in puppies and the ages at which hearing and vision become active in puppies) were determined for the first time not only for the Kars Shepherd dog but also at the level of dog genotypes in Türkiye. In addition, the detailed evaluation of the fertility characteristics of the offspring obtained from the first breeding flock further increases the scientific importance of the study. It is hypothesized that the findings obtained will make significant contributions to existing literature in terms of conservation, breeding and understanding of the genetic diversity of the breed.

## Author Contributions

**Fikret Bektaşoğlu**: investigation, methodology, validation, project administration, data curation, writing – review and editing, writing – original draft, conceptualization, formal analysis, supervision, software, resources. **Turgut Kırmızıbayrak**: methodology, writing – original draft, writing – review and editing, formal analysis, supervision, investigation, conceptualization, project administration, data curation, software, visualization.

## Ethics Statement

The Kafkas University Local Ethics Committee for Animal Experiments (KAÜ‐HADYEK/2021‐146), Kars, Türkiye, gave its clearance before this study could be conducted.

## Conflicts of Interest

The authors declare no conflicts of interest.

## Peer Review

The peer review history for this article is available at https://www.webofscience.com/api/gateway/wos/peer‐review/10.1002/vms3.70660.

## Data Availability

The data that support the findings of this study are available from the corresponding author upon reasonable request.
